# Small-area variation  of cardiovascular diseases and select risk factors and their association to household and area poverty in South Africa: Capturing emerging trends in South Africa to better target local level interventions

**DOI:** 10.1371/journal.pone.0230564

**Published:** 2020-04-22

**Authors:** Ntabozuko Dwane, Njeri Wabiri, Samuel Manda

**Affiliations:** 1 Empilweni Services Research Unit, Department of Paediatrics and Child Health, University of Witwatersrand, Johannesburg, South Africa; 2 Human Sciences Research Council, Pretoria, South Africa; 3 Biostatistics Research Unit, South African Medical Research Council, Pretoria, South Africa; 4 Department of Statistics, University of Pretoria, Pretoria, South Africa; University of Washington, UNITED STATES

## Abstract

**Background:**

Of the total 56 million deaths worldwide during 2012, 38 million (68%) were due to noncommunicable diseases (NCDs), particularly cardiovascular diseases (17.5 million deaths) cancers (8.2 million) which represents46.2% and 21.7% of NCD deaths, respectively). Nearly 80 percent of the global CVD deaths occur in low- and middle-income countries. Some of the major CVDs such as ischemic heart disease (IHD) and stroke and CVD risk conditions, namely, hypertension and dyslipidaemia share common modifiable risk factors including smoking, unhealthy diets, harmful use of alcohol and physical inactivity. The CVDs are now putting a heavy strain of the health systems at both national and local levels, which have previously largely focused on infectious diseases and appalling maternal and child health. We set out to estimate district-level co-occurrence of two cardiovascular diseases (CVDs), namely, ischemic heart disease (IHD) and stroke; and two major risk conditions for CVD, namely, hypertension and dyslipidaemia in South Africa.

**Method:**

The analyses were based on adults health collected as part of the 2012 South African National Health and Nutrition Examination Survey (SANHANES). We used joint disease mapping models to estimate and map the spatial distributions of risks of hypertension, self-report of ischaemic heart disease (IHD), stroke and dyslipidaemia at the district level in South Africa. The analyses were adjusted for known individual social demographic and lifestyle factors, household and district level poverty measurements using binary spatial models.

**Results:**

The estimated prevalence of IHD, stroke, hypertension and dyslipidaemia revealed high inequality at the district level (median value (range): 5.4 (0–17.8%); 1.7 (0–18.2%); 32.0 (12.5–48.2%) and 52.2 (0–71.7%), respectively). The adjusted risks of stroke, hypertension and IHD were mostly high in districts in the South-Eastern parts of the country, while that of dyslipidaemia, was high in Central and top North-Eastern corridor of the country.

**Conclusions:**

The study has confirmed common modifiable risk factors of two cardiovascular diseases (CVDs), namely, ischemic heart disease (IHD) and stroke; and two major risk conditions for CVD, namely, hypertension and dyslipidaemia. Accordingly, an integrated intervention approach addressing cardiovascular diseases and associated risk factors and conditions would be more cost effective and provide stronger impacts than individual tailored interventions only. Findings of excess district-level variations in the CVDs and their risk factor profiles might be useful for developing effective public health policies and interventions aimed at reducing behavioural risk factors including harmful use of alcohol, physical inactivity and high salt intake.

## Introduction

Non-communicable diseases (NCDs) are a major cause of deaths worldwide, with two out of five deaths attributable to NCDs [1]. The costs and consequences related to the burden of the NCDs will be borne largely by developing countries in the future [1]. Even though there is evidence of direct interactions between NCDs and certain infectious diseases (IDs) [2], the emergence of NCDs in developing countries is largely due to the phenomenon of demographic and nutritional transitions. This comes about as a result of changes in lifestyle as populations move to more urbanized patterns [3]. High food prices expose the poor to food insecurity leading to either under or over-nutrition. On the other hand, changes in the food environment are associated with increasing consumption of unhealthy foods that are high in fat, sugar and salt. Unhealthy food uptake, though energy dense, lacks overall nutritional value and has been shown to increase levels of overweight and obesity [4]. At the same time, there is a pervasiveness of other major risk factors for non-communicable diseases such as declining levels of physical activity and physical fitness at a population level and exposure to secondary smoke and smoking is a concern [5]. Likewise, the awareness of these conditions and interventions targeted at their prevention, treatment and control remains very low in sub-Saharan Africa [6].

Scientific and empirical evidence of the association between NCDs, nutritional, and lifestyle risk factors is vast. A systematic review of the socio-demographic patterns of hypertension prevalence in low-and-middle-income countries (LMIC) found that the prevalence of hypertension was associated with and varied by socio-demographic region [7]. Elsewhere, Ford and Highveld [8] have shown that geographic disparities in the distribution of certain cardiovascular morbidity have been observed and linked to socioeconomic deprivation; however, little is known about the geographic overlaps in these conditions. The overlapping epidemiology of the conditions may be better understood by pooling data across the conditions in a unified way [9]. Some limited studies have appeared in this area, for example; Kandala et al [10,11] demonstrated evidence of a geographic distribution of cardiovascular diseases in South Africa mediated by lifestyle-related factors. However, the studies where based on analyses of rather “outdated” 1998 South Africa Demographic and Health Survey (SADHS) data.

South Africa is simultaneously experiencing numerous epidemics of infectious diseases, non-communicable diseases and trauma which are not typical of the phenomenon of epidemiological transition. With the growing epidemic of chronic diseases including CVDs (such as hypertension, stroke and heart disease), metabolic syndrome and diabetes and cancers [12–14], the South African National Department of Health (NDoH) strategies are aimed at reducing NCD morbidity, mortality and associated risk factors [15] have identified CVDs as a priority. To achieve this, an in depth epidemiological investigation is required to identify populations and areas at high risk of exposure to adverse CVD outcomes. This would aid in the implementation of the National NCD strategies and plans. To contribute to these endeavours, this study presents an analysis that makes use of very recent high quality data sources; namely, the 2012 South African National Health and Nutrition Examination Survey (SANHANES-I) [5]. This is the only health and nutrition survey that has been conducted in South Africa and it represents the largest population based health status survey that has been conducted in the country. Other data elements used in the study are based on the official statistics from the Statistics South Africa and include the South Africa Multidimensional Poverty Index (SAMPI)) [16]. These data are triangulated at district level in order to provide a finer spatial resolution not accounted for in the previous analyses.

Within this background, the study aimed to 1) estimate the correlation between two cardiovascular diseases (CVDs), namely, ischemic heart disease (IHD) and stroke; and two major risk conditions for CVD, namely, hypertension and dyslipidaemia in South Africa (adjusting for confounding of known individual socio-demographics and lifestyle risk factors, household district level poverty measures at district level) and 2) estimate and map the smoothed geographic pattern of hypertension, diabetes, CHD, stroke and dyslipidaemia at the district level in South Africa.

## Methods

### Survey sampling, field and laboratory procedures

The study is a secondary analysis of individual-level cardiovascular and risk factors using data from SANHANES-1 [5]. This cross-sectional population-based household survey was conducted in 2012, using a multi-stage disproportionate, stratified cluster sampling approach. Stratification was done by province and locality type, and in the formal urban areas race was also used as a third stratification variable. Sampling frames were based on Enumerator areas (EA) used in the South Africa national census, updated to reflect changes in the socio demographic profile of the country since 2011. A total of 500 EAs were selected from a database of 86,000 EAs as the primary sampling units; 20 households within each selected EA constituted the secondary sampling unit (10,000 households) and all individuals within households formed the final sampling unit. A ‘household’ was defined as a person, or a group of persons, who occupied a common dwelling (or part of it) for at least four days a week and who provided for themselves jointly with food and other essentials for living. In other words, they lived together as a unit (Statistics South Africa (StatsSA)). Persons who occupied the same dwelling, but who did not share food or other essentials, were enumerated as separate households. A ‘household member’ was defined as any person who slept in the household for at least four nights a week. Individuals staying in educational institutions, old-age homes, hospitals, homeless people, and uniformed-service barracks were not eligible to participate in the survey. Further details of the survey design are in [5] (SANHANES) and the data used in this paper is available from (http://curation.hsrc.ac.za/Dataset-565-datafiles.phtml)

Poverty and deprivation data used in our study are contained in a report by Statistics South Africa (Stats SA) [17]. In the report Stats SA show how they conceptualised and constructed the South African Multidimensional Poverty Index (SAMPI) using data collected through the Household and Population censuses of 2001 and 2011. The SAMPI is based on the global Multidimensional Poverty Index (MPI) which is an international measure of acute poverty. The SAMPI compliments traditional income and expenditure-based poverty measures by capturing the severe deprivations that each person or household faces with respect to four dimensions:—education (measured by years of schooling and school attendance indicators); health (measured by nutrition and child mortality indicators); living standards (measured by indicators such as cooking fuel, sanitation, water, electricity, floor, and assets); and economic activity (measured by level of adult unemployment). The SAMPI is the product of the two measures: the headcount (i.e. proportion of households that are multidimensionally poor) and the intensity of poverty (i.e. average proportion of indicators in which poor households are deprived). Example, a headcount poverty of 20% implies that 20% of all households were poor in 2011, and an average intensity of poverty of 44% amongst the poor households (imply that the poor households experienced deprivation on 44% of all the four dimensions in 2011). The SAMPI then equals 0.09 (= 20% x 44%). In an extremely poor society where all households are poor and are deprived in all dimension indicators, the SAMPI score would be 1.0. However, in an impoverished society where 50% of households are poor and experienced deprivation on 50% of all dimensions, the SAMPI score would be 0.25. The SAMPI score ranges from 0 to 1, with 0 indicating least SAMPI poor and 1 indicating the most SAMPI poor district.

### Ethical statement

Study activities were approved by the Human Science Research Council Research Ethics Committee.

### Study measures and outcomes

The study self-reported measures of health and previously diagnosed conditions included a history of diabetes; alcohol use; smoking; high blood pressure, heart disease, stroke, high blood cholesterol and high blood sugar.

### The study outcomes included four individual-level cardiovascular diseases

- High blood pressure (HBP) or hypertension which combined both clinical measurements with the cut-off of systolic (SBP) ≥140mmHg OR a cut-off of diastolic blood pressure (DBP) ≥90mmHg (JNC-7) [21] and/or a self-reported history of high blood pressure, or on medication for high blood pressure. HBP and “hypertension” will be used interchangeably in the paper
- Ischaemic heart disease (IHD) which was measured as self-reported histories of; “heart attack, angina and chest pain” (histories of these symptoms were elicited together in the survey)
- Stroke which was based on a self-reported history of stroke and
- Dyslipidaemia which was measured by high levels of lipids (cholesterol, triglycerides, or both), and abnormally high levels of low density lipoprotein (LDL) and abnormally low levels of high density lipoprotein (HDL), or both. For this study, dyslipidaemia was indicated by use of lipid lowering drugs OR diagnosis of dyslipidaemia OR Total cholesterol > = 6.22mmol// (or 240 mg/dl) a OR Triglycerides > = 1.69mmol/l (or 150 mg/dl); LDL > = 4.14mmol/l (or 160mg/dl) OR HDL <1.04mmol/l (men <40mg/dl) and HDL < 1.29mmol/l (women <50mg/dl) [18,19]

**Individual-level determinants that were modelled** included a number of socio-demographic and socio-economic, lifestyle and co-morbidity factors associated with vascular diseases which were extracted from the data. For instance, respondent’s age at the time of survey was included as an indicator of birth cohort of the participant. Other predictor variables included sex, ethnicity (black/African vs. coloured, white and Asian/Indian), education (no education vs. primary, matric and tertiary education), measured body mass index (BMI) using recommended CDC/WHO cut-offs [20] to ascertain normal weight (BMI 18.5 > 25.0) vs. underweight (BMI < 18.5), overweight/obese(BMI ≥ 25) (, impaired glucose metabolism, smoking (never smoked vs. smoking history and current smoker), drinking status (non-current alcohol vs. current alcohol drinker) and diabetes which was defined as an HbA1C of ≥ 6.5% for impaired glucose homeostasis defined as those with HbA1C ≥ 6.5% and/ or a history of being diagnosed with diabetes and/or currently being on treatment for diabetes.

We also considered a number of contextual factors, including place of residence/ locality [rural (formal/informal) vs. urban (formal/informal)]; household level socio-economic status (wealth quintiles) and district poverty index levels.

### Data weighting

In this study we re-looked at the weighting options of self-report and measured health outcomes. Previously-developed survey weights were applied in order to provide estimates representative of the population. We used differential weighting between those who consented to biomarker and physical measurements and those that did not but provided self-reported health outcomes.

### Spatial unit of analysis

**Fig 1:**
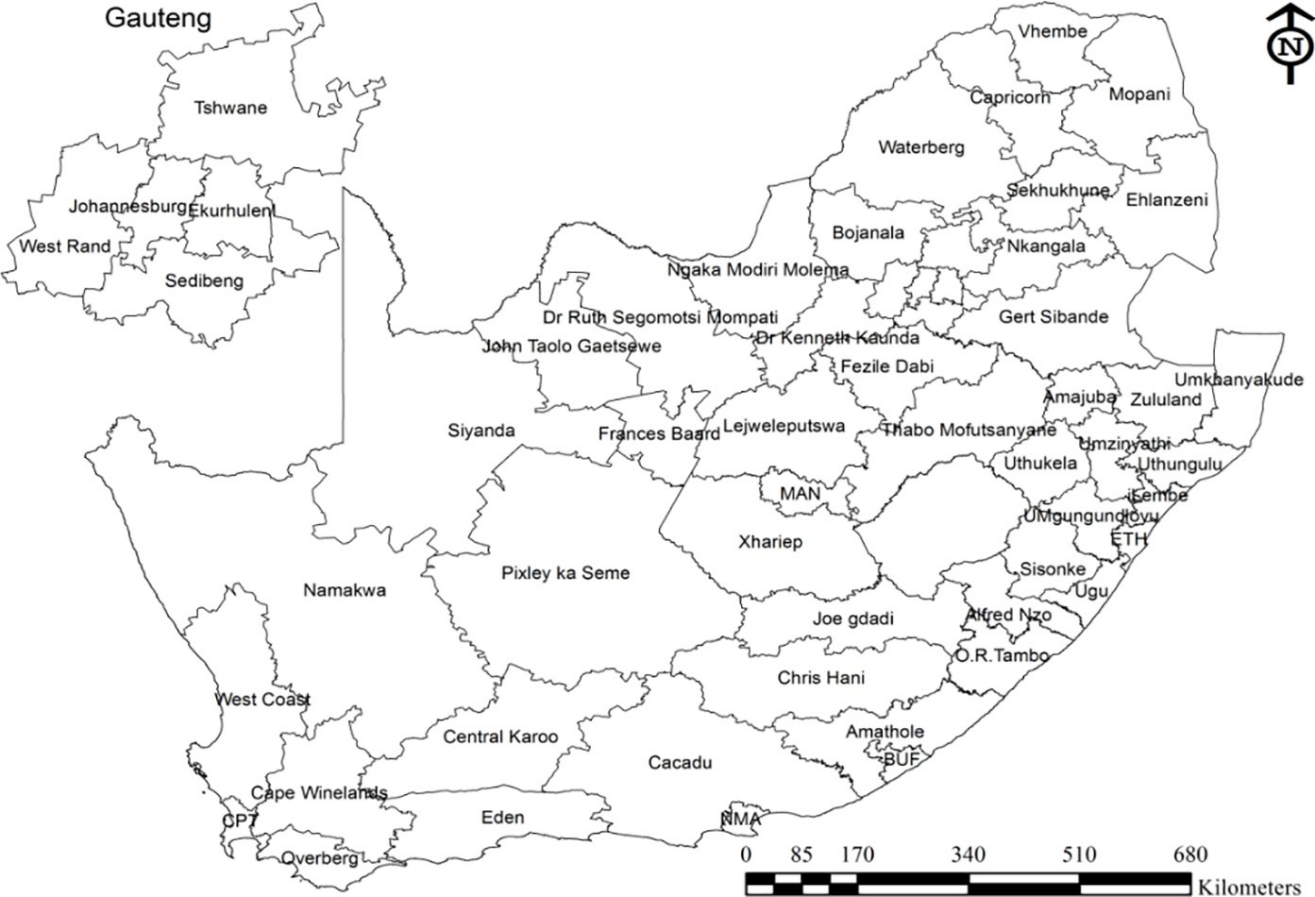
Map of health districts in South Africa.

The District Health System (DHS) is the basic platform from which the delivery of Primary Health Care is provided in South Africa [21]. Currently, there are 52 Health Districts in the country (Fig 1). This is the grouping unit that we used for the spatial modelling in this paper. Preliminary data analyses were done in STATA, and spatial model fitting was carried out using the freely available WinBUGS package. Descriptive maps for this paper were generated by authors using ArcGIS [22] and the fitted smoothed spatial estimates were mapped using GeoBUGS Software [23].

### Modelling geographical co-occurrence of the four conditions

We have indicated earlier that the health conditions considered for disease mapping in the study may share the same set of (both individually and spatially distributed) risk factors, or may be linked etiologically. Furthermore, the presence of one condition might encourage the presence of another over a district, for example high levels of blood cholesterol in a district may lead to high level of ischemic heart conditions. In such situations, it is more appropriate to consider analyzing the spatial epidemiology of the four health conditions using multivariate spatial modeling. We could have opted to use four separate univariate spatial models. However, because correlation across the four diseases may occur, multivariate spatial model was used to take into account the dependence structure. The use of joint spatial models permitted modeling of dependence among those four conditions while maintaining spatial dependence between districts. Identifying similar patterns in geographical variation of the four possibly related conditions in a multivariate model potentially provides more convincing evidence of any real clustering of underlying risks than would otherwise have been possible using the analysis of any single disease separately.

A number of multivariate mapping models have been proposed in the literature including the shared spatial component models [24,25] and multivariate conditionally autoregressive (MCAR) models [26–28]. These joint mapping models have been used to model and estimate risks of related cancers [29–31] childhood illnesses [32] and childhood cancer and diabetes [33,34]. We suppose *Y*_*ijk*_ is a binary response corresponding to participant *i* in district *j* having a *k* CVD disease *k* (*j* = 1,…,52; *i* = 1,…,*N*_*i*_; *k* = 1,…,4) taking values of 1 if the participant has had the disease and 0 otherwise. Furthermore, suppose *X*_*ij*_ is the vector of covariates associated with subject *ij*. The covariates *X*_*ij*_ are used to explain variations in area disease risk through the associated disease-specific fixed effects parameters *β*_*k*_. For each district, we also model unobserved disease-specific district-spatial variation *U*_*jk*_. If there are no covariates to model the spatial patterning of risks then this represents spatial patterns in the disease-specific outcome; otherwise it captures disease-specific spatial structure in the residuals. By taking the binary responses *Y*_*ijk*_ as independent Bernoulli random variables with parameters *π*_*ijk*_ being the probability of subject *ij* having disease *k*, we have

$$log\left(\frac{\pi_{ijk}}{1-\pi_{ijk}}\right)=\alpha_{k}+{\beta \prime }_{k}X_{ij}+U_{jk}$$

where *α*_*k*_ is disease *k* constant term.

The excess spatial variations *U*_*jk*_ often show spatial correlation and disregarding them in the modelling and estimation procedures can lead to biased and inefficient inference [35].

There are various ways of modelling the spatial effects. The convolution model, which involves decomposing the spatial variations *U*_*jk*_ as the sum of two sets of random effects: a spatially structured effects *S*_*jk*_, that represents spatially correlated risks and is distributed as a conditional autoregressive (CAR) model and the other the unstructured parts are distributed as independent and identically random effects, *V*_*jk*_ is used here [36]. The four district-level spatially structured effects were assumed to arise from a multivariate CAR distribution with precision matrix $\Sigma_{s}^{-1}$, which was assumed to follow a Wishart distribution. To represent vague prior knowledge, we chose the degrees of freedom for this distribution to be as small as possible (i.e. 4, the rank of Σ_*s*_). The four unstructured spatial random effects were modelled as multivariate normal with covariance matrix Σ_*v*_ with $\Sigma_{v}^{-1}$ taken to be Wishart distributed with 4 degrees of freedom and appropriate scale matrix. This is different from the methods used in [10,11, 30–34] where shared spatial component models were employed.

The parameters *α*_*k*_′*s* and *β*_*k*_′*s* are the disease-specific baseline risk and fixed effect risks associated with the risk vector *X*_*ij*_. For a Bayesian model to be complete, all unknown parameters, whether for fixed or random effects, are given prior distributions. Since we are using a CAR Normal prior, with sum-to-zero constraints on the random effect terms, we assigned a flat prior on the disease-specific risk terms, *α*_*k*_ and the fixed effects were assigned independent *Normal* (0, 10^3^) prior distributions. The precision matrices Σ^−1^ for the multivariate models were assigned a *Wishart* prior distributions as in [34].

## Results

### Study sample

Of the 10 000 households or visiting points (VPs) sampled, 8 168 were valid, occupied households and 1 832 were invalid or clearly abandoned. Of the 8 168 valid VPs/ households, 6 306 (77.2%) were interviewed and 1 289 (15.8%) refused to take part in the survey. Of the 8 168 valid households that agreed to participate in the survey, 27 580 individuals were eligible to be interviewed. A total of 25 532 individuals 92.6%)/ completed the interview whilst 7.4% refused to participate. The 25 532 individuals who agreed to be interviewed were further invited to participate in the physical and clinical examination conducted in the mobile clinics. Nearly 17,000 adults (age ≥ 15 years) were examined.

A summary of cardiovascular disease data and their associations with participant level socio-demographic, BMI and lifestyle risk factors from univariate analysis are provided in Table 1.

**Table 1:** Survey weighted prevalence of risks of cardiovascular disease by socio-demographic factors, BMI and smoking.

	High Blood Pressure(HBP)	Ischaemic Heart Disease(IHD)	Stroke	Dyslipidaemia	Total
	%(95% CI)	%(95% CI)	%(95% CI)	%(95% CI)	%(N)
Total	28.6 (27.0 30.3)	5.4 (4.7–6.1)	1.8(1.5–2.1)	53.1(50.6–55.6)	16293
***Sex***					
Males	24.1(22.1–26.2)	4.5(3.6–5.5)	1.7(1.3–2.2)	47.7(43.8–51.6)	41.7(39.4–44.0)
Females	32.6(30.7–34.4)	6.1(5.4–7.0)	1.9(1.6–2.3)	57.8(55.3–60.2)	58.3(56.0–60.6)
***Age***					
15–24	8.8(7.1–10.9)	2.2(1.6–3.0)	0.4(0.2–0.7)	39.9(35.9–44.1)	17.7(15.8–19.9)
25–34	12.9(10.9–15.3)	3.8(3.0–4.9)	0.8(0.5–1.2)	54.8(48.5–61.0)	15(13.1–17.3)
35–44	24.1(21.6–26.7)	5.6(4.4–7.2)	1.5(1.0–2.2)	55.3(49.9–60.6)	14.9(13.3–16.6)
45–54	44.2(40.4–48.2)	7.8(6.2–9.8)	2.4(1.8–3.2)	63.9(59.4–68.1)	19.6(17.5–21.8)
55–64	58.6(53.5–63.5)	10.1(8.1–12.6)	4.5(3.2–6.2)	60.0(54.0–65.8)	17.1(15.2–19.2)
65+	69.9(65.9–73.6)	11.4(8.4–15.3)	6.8(4.8–9.4)	61.3(55.9–66.4)	15.6(13.8–17.5)
***Education***					
No school	47.9(43.7–52.2)	9.5(7.2–12.5)	3.1(2.1–4.5)	57.9(51.4–64.0)	10.3(8.0.7–12.2)
Primary	43.0(38.9–47.3)	9.5(7.6–11.6)	3.4(2.5–4.4)	55.2(51.3–59.1)	23.6(21.4–26.0)
Secondary	24.3(22.1–26.7)	4.9(4.0–6.0)	1.9(1.5–2.5)	53(49.2–56.7)	35.7(33.3–38.1)
Matric	19.9(17.7–22.4)	4.3(3.4–5.4)	1.1(0.7–1.7)	45.9(40.7–51.3)	20.5(18.2–23.1)
Higher Education	26.3(21.9–31.2)	3.5(2.1–5.6)	0.9(0.5–1.6)	56.7(46.0–66.9)	9.9(7.9–12.4)
***Locality***					
Urban formal	30.2(27.7–32.9)	5.1(4.1–6.2)	1.6(1.3–2.1)	54.7(50.6–58.6)	55(48.8–61.1)
Urban informal	22.2(19.3–25.4)	5.3(3.8–7.5)	1.6(1.0–2.7)	49.6(41.5–57.8)	8.5(5.9–12.0)
Rural tribal Areas	27.9(25.4–30.6)	6.4(5.3–7.8)	2.3(1.7–3.0)	53.3(49.5–57.1)	28.6(23.6–34.1)
Rural formal (Farms)	27.2(23.2–31.7)	4.1(3.0–5.5)	1.7(0.9–2.9)	47.7(42.0–53.5)	7.9(5.6–11.2)
***Province***					
Western Cape	36.3(33.0–39.8)	3.8(2.8–5.2)	3.5(2.4–5.0)	55.8(50.8–60.7)	11.9(10.3–13.7)
Eastern Cape	32.3(28.8–35.9)	7(5.2–9.2)	1.5(1.0–2.3)	56.5(51.4–61.5)	11.4(9.3–13.8)
Northern Cape	30.3(25.7–35.4)	5.2(3.4–8.0)	2.4(1.4–4.0)	56(48.3–63.4)	2.2(1.6–2.8)
Free State	38.8(34.7–43.0)	11.7(8.4–15.9)	1.3(0.7–2.3)	50.3(45.4–55.2)	5.9(4.4–7.9)
Kwa-Zulu Natal	31.4(28.3–34.6)	7.8(6.3–9.7)	3.1(2.3–4.1)	61.7(56.4–66.6)	20.6(16.8–25.0)
North West	26.4(22.0–31.2)	4.4(3.1–6.4)	1.9(1.2–3.2)	46.2(42.5–50.0)	6.6(5.4–8.0)
Gauteng	25.9(21.9–30.4)	3.7(2.5–5.5)	0.6(0.4–1.0)	50.9(43.7–58.0)	27(21.4–33.6)
Mpumalanga	20.0(15.8–24.8)	5.3(3.6–7.7)	3.5(2.0–6.1)	41.6(34.6–49.0)	4.9(3.5–6.8)
Limpopo	20.7(17.5–24.3)	4.3(3.0–6.0)	1.2(0.7–2.2)	50.1(42.9–57.3)	9.5(7.6–11.8)
***Race***					
African	27.5(25.6–29.6)	5.6(4.8–6.6)	1.6(1.3–2.0)	51.1(48.5–53.6)	79.4(76.0–82.5)
White	27.7(21.6–34.8)	4.0(2.5–6.4)	2.3(1.2–4.1)	73.4(57.6–84.8)	6.5(4.7–9.0)
Coloured	37.6(35.0–40.4)	4.2(3.1–5.6)	2.6(2.0–3.4)	54.0(50.0–57.9)	11(9.2–13.2)
Indian/Asian	28.8(23.3–35.1)	6.5(4.5–9.3)	1.7(0.9–2.9)	67.1(57.0–75.8)	2.9(2.1–4.0)
Other	51.1(16.7–84.4)	7(2.2–19.9)	0	16.0(3.9–47.0)	0.2(0.1–0.5)
***BMI***					
Underweight	21.6(17.4–26.5)	4.9(3.1–7.5)	1.8(0.9–3.5)	33.7(27.9–39.9)	5.1(4.2–6.1)
Normal	26.8(23.8–30.0)	5.8(4.5–7.3)	1.3(1.0–1.9)	42.7(38.5–46.9)	35.7(32.9–38.6)
Overweight	48.3(45.5–51.0)	9(7.6–10.6)	3.3(2.6–4.3)	65.8(62.8–68.8)	59.2(56.3–62.0)
***Smoking***					
Never	26.9 (25.1–28.8)	5.1(4.5–5.9)	1.6(1.3–1.9)	52.4(49.7–55.1)	77.5(75.4–79.5)
Ex-smoker	43.9(35.1–53.2)	5(3.9–6.4)	2.3(1.6–3.3)	58.1(43.4–71.4)	18.9(17.0–20.9)
Current	31.2(28.2–34.3)	14.7(9.7–21.7)	5(2.9–8.2)	49.2(44.2–54.3)	3.6(2.9–4.4)
***Alcohol use***					
Never	28.4(26.6–30.3)	5.5(4.7–6.3)	1.9(1.6–2.3)	53.5(50.8–56.2)	75.6(73.0–78.1)
Alcohol	27.7(25.0–30.6)	5.1(4.2–6.3)	1.7(1.3–2.3)	47.9(43.3–52.4)	24.4(21.9–27.0)
Diabetes*					
No	24.7(23.0–26.5)	4.7(4.1–5.4)	1.5(1.3–1.9)	50.2(47.8–52.7)	85(83.1–86.7)
Yes	72.6(67.6–77.0)	14.9(11.8–18.7)	6(4.2–8.6)	74.5(69.4–79.0)	15(13.3–16.9)
***Household Wealth Quantiles***					
Lowest(QI)	26.7(24.0–29.5)	6.3(5.0–8.0)	1.5(1.1–2.1)	53.5(49.1–57.7)	21(17.4–25.0)
QII	28.8(26.2–31.5)	5.7(4.6–7.2)	2.1(1.6–2.8)	52(47.5–56.5)	19.1(16.7–21.8)
QIII	28.3(25.4–31.4)	6.5(5.3–7.9)	2(1.4–2.9)	50.5(46.1–54.8)	20.5(17.7–23.7)
QIV	32.6(29.3–36.2)	5.3(4.2–6.7)	2.2(1.5–3.2)	52.3(47.2–57.4)	20.6(17.7–23.9)
Highest(QV)	27.4(23.9–31.3)	3.8(2.7–5.4)	1.4(0.9–2.1)	55.6(49.5–61.6)	18.8(16.1–21.8)

* normal weight (BMI 18.5 > 25.0) underweight (BMI < 18.5), overweight/obese(BMI ≥ 25)

An overall total of 16293 adults (6729 males and 9564 females) reported cardiovascular conditions. Of these adults, 28.6% (CI 27.0%-30.3%) had hypertension, 5.4% (CI 4.7%-6.1%) had a history of ischaemic heart disease, 1.8% (1.5%-2.1%) had a history of stroke and 53.1% (CI 50.6%-55.6%%) had dyslipidaemia. High blood pressure (HBP) and dyslipidaemia were the most prevalent cardiovascular conditions. There were no significant differences in prevalence of all of these diseases between males and females. Participants with stroke were significantly more likely to also have high blood pressure (whether self-reported or measured). Furthermore, having IHD was linked to having had HBP. We also computed sensitivity and specificity of self-reported conditions using the measured conditions as gold standards. Both self-reported high blood pressure and dyslipidaemia, at 44.6% and 6.6%, had very low sensitivity values but very high specificity values at 85.1% and 97.9% respectively.

### Association of high blood pressure (HBP) with individual risk factors

The prevalence of HBP increased significantly with increasing age with the majority of the population over 45 years being affected (44.2%-69.9%). The prevalence of HBP also decreased with increase in levels of education to a low of 19.9% (CI17.7%-22.4%%) among those with a matric level of education, but increased among those with education above matric to 26.3%(CI(21.9%-31.2%). Those with no schooling also had significantly high levels of HBP (47.9%; CI 43.7%-52.2%) compared with all other education levels. In terms of household wealth quintiles, though those in lower quintiles had lower rates of HBP than those in high quintiles, however, the differences were not significant. Almost one third (30.2%; CI 27.7%-32.9%) of people living in urban formal areas had a higher prevalence of HBP compared to approximately a quarter of those living in urban informal areas (22.2%; CI 19.3%-25.4%).

Geographically, the lowest prevalence of HBP (≤20%) was reported in districts in Limpopo province. Significant differences in prevalence were noted between population groups with Coloured the most affected by HBP (37.6%; CI 35.0%-40.4%) compared to 27.5%; (CI 25.6%-29.6%) of the Black African population group. Approximately half of obese individuals (48.3%; CI 45.5–51.0) had significantly higher HBP in comparison to approximately a fifth of underweight individuals (21.6%; CI 17.4%-26.5%)There was a significant difference in the prevalence of HBP found between those with a history of smoking which included current smokers and those that had never smoked. No significant difference in the prevalence of HBP was found between people reporting alcohol use and people who had never consumed alcohol. The prevalence of HBP among those with diabetes (72.6%; CI 67.6%-77.0) was approximately three times the prevalence of HBP among those who were not diabetic (24.7%; CI 23.0–26.5).

### Association of ischaemic heart disease (IHD) with individual risk factors

The prevalence of IHD increased with increasing age following a similar pattern to the prevalence of hypertension but with a lower prevalence (2.2% - 11.4%) and an earlier plateau in the 55–64 age group. Significant differences in prevalence were noted by levels of education. People with higher levels of education had the lowest levels of IHD (3.5%; CI 2.1%-5.6%). The prevalence among those with no schooling (9.5%; CI 7.2–12.5%) or primary school education (9.5%; CI 7.6%-11.6%)%) was significantly higher compared to those in higher education groups by three-fold. No significant differences were found in the prevalence of IHD between population groups, localities and wealth quintiles. A similar pattern of prevalence to that seen for hypertension by BMI was observed for IHD. Those who reported currently smoking had significantly higher risk of IHD (14.7%; CI 9.7%-21.7%), 3 times higher than previous (5.0%; CI 3.9%-6.4%) and never smoked (5.1%; CI 4.5–5.9%). There was a significant difference between those reporting alcohol use and those who never used alcohol The prevalence of diabetes was 3 times higher among those with IHD, a similar patter observed for the HBP.

### Association of dyslipidaemia with individual risk factors

Approximately 53.1% (50.6–55.6%) (Table 1) of adults had dyslipidaemia, while over half of adults 25 years and older reported dyslipidaemia. The highest prevalence was found among the adults 45-54years and older (63.9%; CI 59.4–68.1%). The lowest prevalence of dyslipidaemia was found among people with a Matric level of education (45.9%; CI 40.7–51.3%); in those with no schooling (61.3%; CI 55.5%-66.9%) and among those with primary school education (56.2%; CI51.8%-60.4%). People with no schooling (57.9%; CI 51.4%-64.0%) and higher education (56.7%; CI 46.0%-66.9%) had significantly higher rates of dyslipidaemia (Table 1).

People in urban informal and rural informal settlements were at significantly lower risk of dyslipidaemia (49.6%; CI 41.5–57.8%) and (47.7%; CI 42.0%-53.5%) respectively than those living in urban formal areas (54.7%; CI 50.6%-58.6%).

Black Africans had amongst the lowest levels of dyslipidaemia (51.1%; CI 48.5%-53.6%), which were significantly lower than those of the other race groups. There was also significant differences in the prevalence of dyslipidaemia among different BMI categories. Those in the overweight population had significantly higher levels of dyslipidaemia (65.8%; CI 62.8%-68.8%) compared to those that were underweight or with normal BMIs. No significant differences were noted between smoking and alcohol use categories although overall the prevalence was high (approximately over 47%).

### Association of stroke with individual risk factors

Of the four cardiovascular conditions, stroke had the lowest prevalence (1.8%; CI 1.5%-2.1%). The rates of stroke increased by approximately 3 times between ages 15–24 (0.4%; CI 0.2%-0.7%); ages 35–44 years (1.5%; CI 1.0%-2.2%) and ages 55–64 (4.5%; CI 3.2%-6.2%)). The lowest prevalence of stroke was found amongst those with Matric (1.1%; CI 0.7%-1.7%) and those with a higher education (0.9%; CI 0.5%-1.6%). These rates more than doubled among those with no education (3.1%; CI 2.1%-4.5%) or primary school (3.4%; CI 2.5%-4.4%). No significant differences in the prevalence of stroke were noted by locality or by race.

Overweight individuals had a significantly higher prevalence of stroke (3.3%; CI 2.6%-4.3%) than individuals with a normal BMI. Current smokers (5.0%; CI 2.9–8.2%) and those with a smoking history (2.3%; CI 1.6%-3.3%) had a higher prevalence of stroke than those who had never smoked (1.6%; CI 1.3%-1.9%). Overall there was a relatively low prevalence of stroke (<2.0%) in most regions across the country compared to the other conditions. Gauteng (0.6% CI 0.4%-1.0%) had the lowest prevalence of stroke. The Western Cape had the highest prevalence of stroke (3.5% CI 2.4%-5.0%), the prevalence of stroke did not differ by household wealth quintiles.

### Health district variation in the prevalence rates

**Fig 2:**
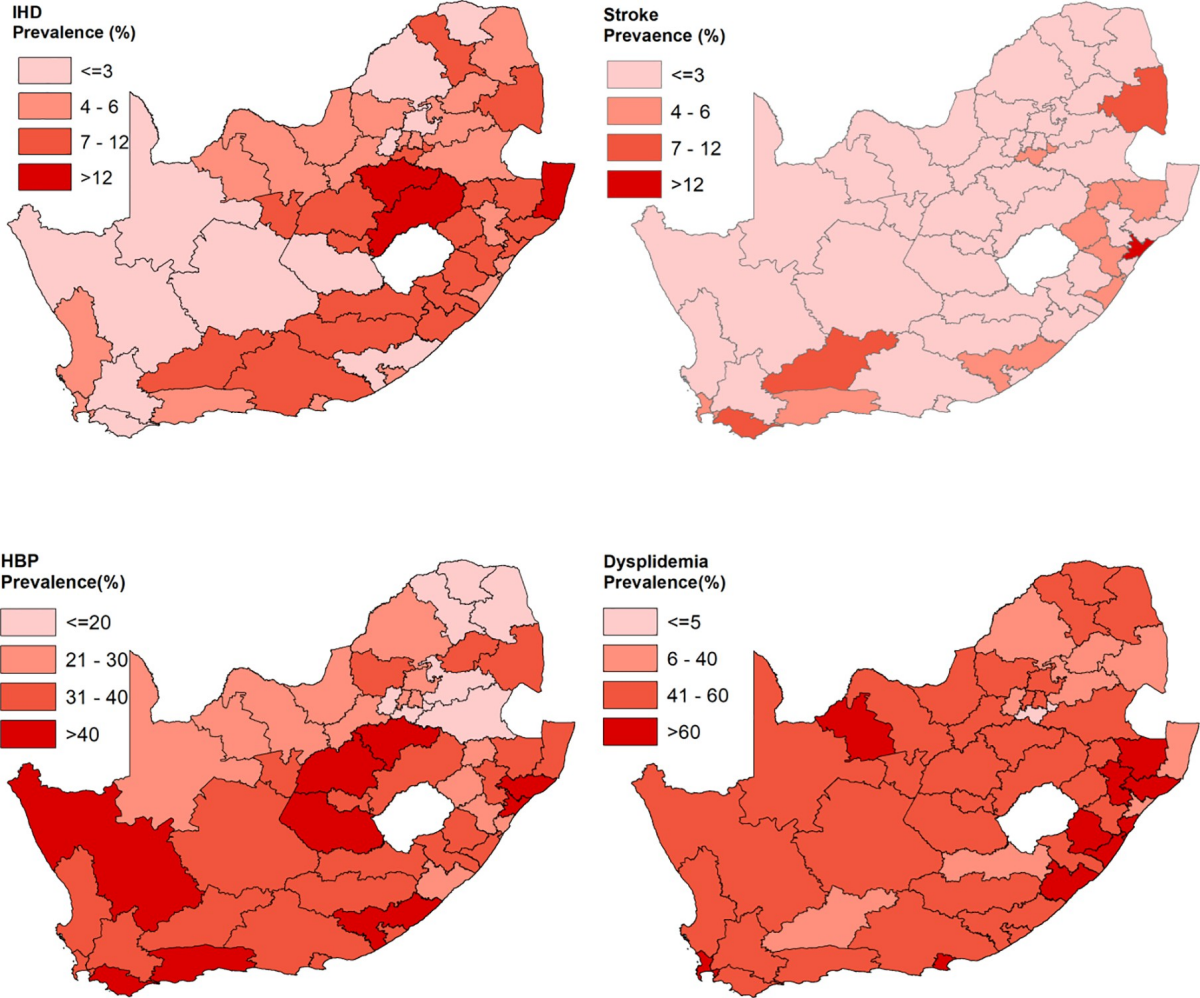
Survey weighted prevalence of risks of cardiovascular diseases by Health District, South African National Health and Nutrition Examination Survey (SANHANES 2012) (Source: Authors).

The sampled participants per district had a *mean* of 346, *median* of 277 and *ranged* from 22-1625.This indicates high variability in the sample sizes. Fig 2 shows prevalence of risks of cardiovascular diseases by Health District from the South African Health and Nutrition Examination Survey (SANHANES 2012). The district level prevalence of HBP had a *mean* of 31.4% and a median value of 32.0%, and a *range* of 12.5%-48.2%. For IHD the *mean* was 6.4% and the *median* was 5.4% with a *range* of 0%-17.8%. For stroke the *mean* was 2.6% and the *median* was 1.7% with a *range* of 0%-18.2. For dyslipidaemia the *mean* was 49.6% and the *median* was 52.2% with a *range* of 0%-71.7%. This shows high instability in the prevalence estimates which might be due to small numbers sampled in some districts.

The disease-specific prevalence maps below show a large amount of noise especially for the rare diseases (stroke and IHD), making it difficult to discern any geographical trends in prevalence. Nonetheless, higher prevalence rates of hypertension were found in the districts of the south-western parts of the country and the lower in the north-eastern parts. Higher prevalence rates of IHD were mostly observed in north-eastern parts, with lower rates in western districts. The distribution of stroke and dyslipidaemia’ prevalence rates appear to be evenly distributed across the country.

### Spatial distribution of district level risk factors

**Fig 3:**
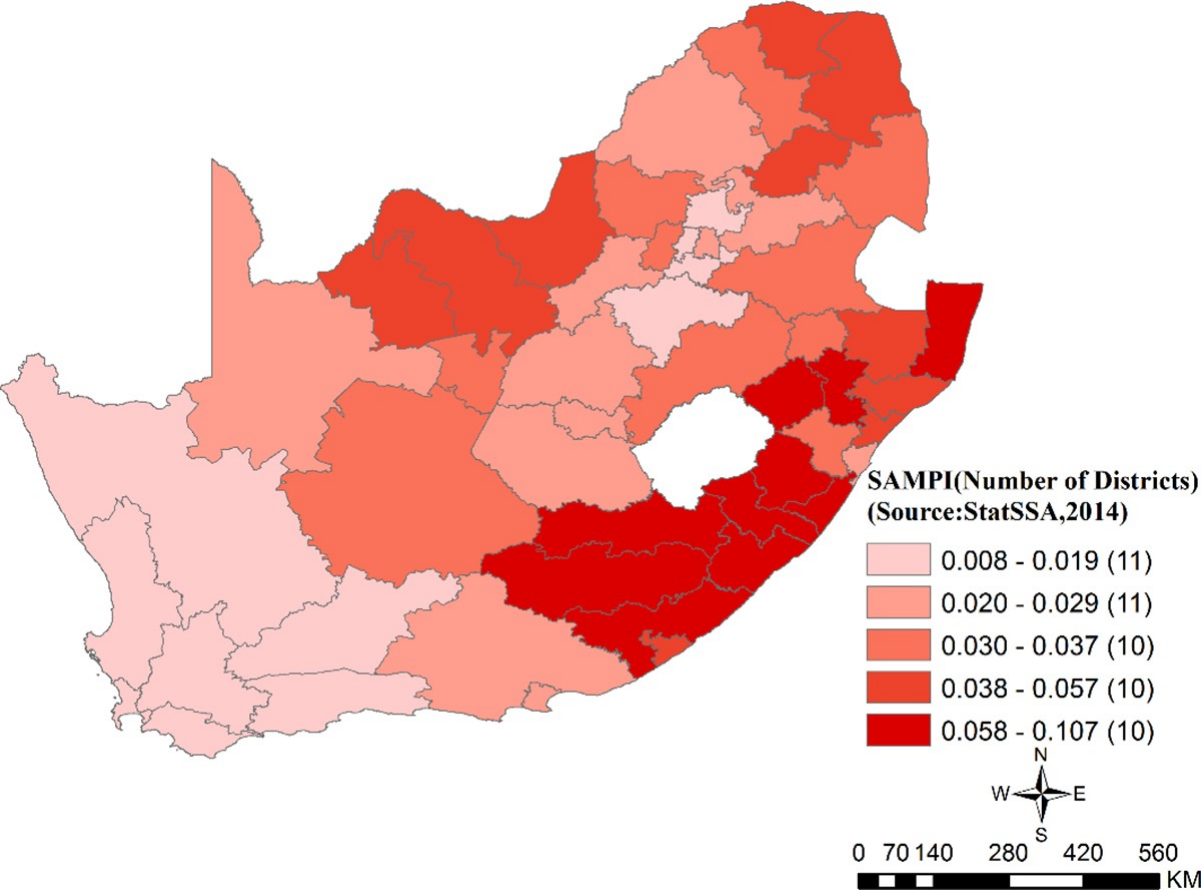
South African Multidimensional Poverty Index (SAMPI) (StatsSA, 2014, Census 2011) (Author generated).

**District socio-economic status measured by South Africa Multidimensional Poverty Index (SAMPI) (StatsSA, 2014).** Fig 3 shows the distribution of SAMPI across the 52 district in South Africa. The most SAMPI poor district, Alfred Nzo (SAMPI score = 0.1) had the highest proportion (25.6%) of poor households which experienced deprivation on 42% of all dimensions. The West Coast district had the least SAMPI poor score of 0.008, with only 2% poor households experiencing deprivation of 42% on all the five dimensions. The 10 most SAMPI poor (SAMP I≥ 0.06) health districts are in the Eastern Cape, KwaZulu Natal, Free State, and Limpopo provinces while the least SAMPI poor (SAMPI ≤ 0.02) health districts include all metropolitan districts and all districts in Gauteng and the Western Cape province.

### Statistical model results

#### Adjusted effects on CVDs using individual and household risk factors

We first fitted the spatial models described above using only subject or individual level risk factors and survey derived household wealth quintiles. The results are shown in the S1 Table. Sex was not associated with a higher risk of cardiovascular disease. Increasing age was positively associated with a higher risk of HBP in the age groups 25–34 years (OR 1.7; CI 1.3–2.3) and above up to age group 45–54 years (OR 11.8; CI 9.0–15.8) compared to age group 15–24 years (OR 23.9; CI 17.4–32.8). Increasing age was also found to be positively associated with increasing risk of dyslipidaemia. It was found to be three to four times higher in older age groups compared to the 15–24 years group. Risk of HBP increased with increasing levels of education compared to those with no schooling though this was not significant. HBP was twice as likely to be associated with being overweight (OR 1.8; CI 1.5–2.2) compared to having a normal weight. The risk for dyslipidaemia was reduced in the underweight category (OR = 0.7; CI 0.5–1.0) and it was positively associated with being overweight (OR 1.6; CI 1.4–1.9) compared to having normal weight. Compared to the risk in 15–24 year olds, the risk of stroke in older age groups was almost five times higher between ages 35–45 (OR 4.6; CI 2.0–11.9); and between 45–54 years (OR 4.8; CI 2.0–12.5). The odds ratio of stroke continued to increase by more than 10 fold after age 65 years (OR 11.7; CI 4.9–30.2). The risk of stroke was also higher among smokers compared to those who never smoked (OR 1.27; CI 0.79–2.02). Being diabetic was associated with increased risk of HBP (OR 2.8; CI 2.20–3.6); IHD (OR 1.8; CI 1.3–2.4) and dyslipidaemia (OR 1.7; CI 1.3–2.1) but not with stroke, though there was a positive association (OR 1.37; CI 0.86–2.15).

Using the individual and survey derived household (wealth quintiles) covariates modelled in Table 2, the covariate-adjusted smoothed estimated spatial odds for each of the four disease-specific risks are shown in S1 Fig. This represents the odds of having a CVD divided by not having a CVD because of being in a given location. The spatial odds range from zero (event will never happen) to infinity (event is certain to happen). Most of the variations in the four diseases appeared to be removed, except for HBP which still had a higher prevalence in some districts and provinces with higher excess risk in south-eastern parts.

**Table 2:** Posterior median odds ratios (95% CI) of the four cardiovascular conditions (HBP, IHD, stroke and dyslipidaemia) across selected individual, household risks including the district level SAMPI poverty level, South Africa 2012.

	High Blood Pressure	Ischaemic Heart Disease	Stroke	Dyslipidaemia
Population Group	Adjusted Odds ratios (95% CI)	Adjusted Odds ratios (95% CI)	Adjusted Odds ratios (95% CI)	Adjusted Odds ratios (95% CI)
**Sex**				
Female	1.00	1.00	1.00	1.00
Male	0.8 (0.71.0)	0.7 (0.5–0.9)	0.9 (0.6–1.3)	0.817 (0.7–0.9)
Age(Years)				
15–24	1.00	1.00	1.00	1.00
25–34	1.7 (1.3–2.2)	2.2 (1.3–3.6)	2.1 (0.8–5.3)	1.141 (0.9–1.4)
35–44	3.8 (3.0–4.9)	3.3 (2.1–5.4)	4.5 (2.1–10.7)	1.313 (1.1–1.6)
45–54	9.1 (7.0–11.7)	3.5 (2.2–5.6)	4.5 (2.1–11.0)	1.609 (1.3–2.0)
55–64	16.2 (12.2–21.2)	4.3 (2.8–7.0)	7.6 (3.5–18.5)	1.483 (1.18–1.9)
65+	26.0 (19.1–35.2)	5.1 (3.2–8.2)	11.1 (4.9–26.6)	1.3 (1.0–1.7)
**Education**				
No schooling	1.00	1.00	1.00	1.00
Primary school	1.1 (0.9–1.5)	0.9 (0.6–1.4)	1.1 (0.6–2.0)	1.2 (0.9–1.5)
Some Secondary school	1.1 (0.8–1.4)	0.8 (0.5–1.2)	1.2 (0.6–2.2)	1.2 (0.9–1.6)
Matric	1.0 (0.7–1.4)	0.5 (0.3–0.9)	0.9 (0.4–2.0)	1.1 (0.8–1.5)
Higher education	1.2 (0.8–1.8)	0.7 (0.4–1.3)	1.3 (0.5–3.1)	0.9 (0.6–1.3)
**Race**				
African	1.00	1.00	1.00	1.00
White	0.9 (0.6–1.6)	1.6 (0.8–3.2)	0.9 (02–2.6)	3.0 (1.9–4.7)
Coloured	1.3 (1.0–1.7)	0.7 (0.5–1.1)	1.1 (0.-1.9)	1.0 (0.8–1.2)
Indian/Asian	0.7 (0.4–1.0)	0.7 (0.4–1.3)	0.4 (0.1–1.0)	1.4 (1.0–2.0)
**Locality**				
Urban formal	1.00	1.00	1.00	1.00
Urban informal	0.8 (0.6–1.1)	0.5 (0.3–0.8)	0.7 (0.3–1.5)	0.8 (0.6–1.0)
Rural informal(Tribal)	0.7 (0.5–1.0)	0.8 (0.6–1.3)	1.2 (0.7–2.1)	0.9 (0.7–1.1)
Rural formal(Farms)	0.9 (0.7–1.2)	0.6 (0.4–1.0)	1.0 (0.5–1.8)	1.0 (0.8–1.3)
**BMI**				
Normal	1.00	1.00	1.00	1.00
Underweight	0.7 (0.5–0.9)	1.3 (0.8–2.0)	1.1 (0.5–2.2)	0.6 (0.5–0.8)
Overweight	1.7 (1.4–2.0)	1.1 (0.8–1.5)	1.1 (0.7–1.7)	1.9 (1.-2.)
**Diabetes**				
**No**	1.00	1.00	1.00	1.00
**Yes**	2.8 (2.2–3.6)	1.8 (1.3–2.4)	1.3 (0.8–2.1)	17 (1.3–2.1)
**Smoking**				
Never	1.00	1.00	1.00	1.00
Smoker	1.0 (0.9–1.3)	1.0 (0.7–1.4)	1.0 (0.6–1.6)	1.0 (0.9–1.2)
**Alcohol use**				
Never	1.00	1.00	1.00	1.00
Drinker	1.1 (1.0–1.3)	1.0 (0.7–1.3)	1.3 (0.8–2.0)	0.8 (0.6–0.9)
**Wealth quintiles (households survey)**				
QI(Lowest)	1.00	1.00	1.00	1.00
QII	1.0 (0.8–1.2)	0.8 (0.6–1.2)	1.2 (0.6–2.3)	0.8 (0.7–1.0)
QIII	0.9 (0.7–1.2)	1.7 (1.2–2.5)	2.3 (1.3–4.3)	0.7 (0.6–0.9)
QIV	0.9 (0.7–1.2)	1.1 (0.8–1.7)	2.1 (1.1–4.1)	0.9 (0.7–1.1)
QV(Highest)	1.2 (0.9–1.7)	0.8 (0.5–1.3)	0.9 (0.4–2.1)	1.4 (1.1–1.8)
SAMPI*				
SAMPI1(Least deprived)	0.8 (0.3–1.6)	1.5 (0.5–4.2)	1.5 (0.6–3.9)	1.0 (0.6–1.6)
SAMPI2	1.1 (0.5–2.3)	2.1 (0.8–5.7)	0.8 (0.3–2.0)	1.2 (0.7–1.9)
SAMPI3	1.1 (0.6–2.0)	1.8 (0.8–4.2)	1.3 (0.6–2.7)	0.9 (0.6–1.4)
SAMPI4	1.2 (0.7–2.3)	1.5 (0.7–3.5)	1.3 (0.6–2.7)	0.9 (0.6–1.3)
SAMPI 5(Most deprived)	1.00	1.00	1.00	1.00

#### Adjusted effects on CVDs using individual, household, and district level SAMPI poverty index risks

The previous sections’ analyses were extended to include district level South African Multidimensional Poverty Index (SAMPI), thought to be associated with the four studied cardiovascular conditions. The results are shown in Table 2. As seen before, sex was not associated with a higher risk of CVD. The risk of HBP was over four times higher in older age groups compared to the 15–24 years age group, OR 1.84 (CI 0.6–5.2) in 25–34 years; OR 4.62 (CI 2.0–11.7) in 35–44 years and OR 11.63 (CI 5.1–30.2) in 65+ years age group. Similarly, increasing age was also found to be positively associated with increasing risk of dyslipidaemia, which was highest among the 55–64 years age group (OR 3.7, CI 2.9–4.8). Risk of HBP increased with increasing levels of education (compared to those with no schooling) among those with primary school education (OR 1.1; CI 0.8–1.6) up to those with higher education (OR 1.3; CI 0.8–2.1). HBP was twice as likely to be associated with being overweight (OR 1.9; CI 1.5–2.2) compared to having normal weight. Risk for dyslipidaemia was reduced in the underweight category (OR 0.7; CI 0.6–0.98) and it was positively associated with being overweight (OR 1.64; CI 1.4–1.91) compared to those of normal weight. As before, being diabetic was associated with increased risk of HBP (OR 2.8; CI 2.2–3.6), IHD (OR 1.8; CI 1.3–2.4) and dyslipidaemia (OR 1.7; CI 1.3–2.1) and with stroke it was also positively associated. (OR = 1.3; CI 0.8–2.1). Those in a least SAMPI poor district were four times less likely to have reported a history of IHD (OR 0.2; CI 0.1–0.6) compared to those in most SAMPI poor districts.

**Fig 4:**
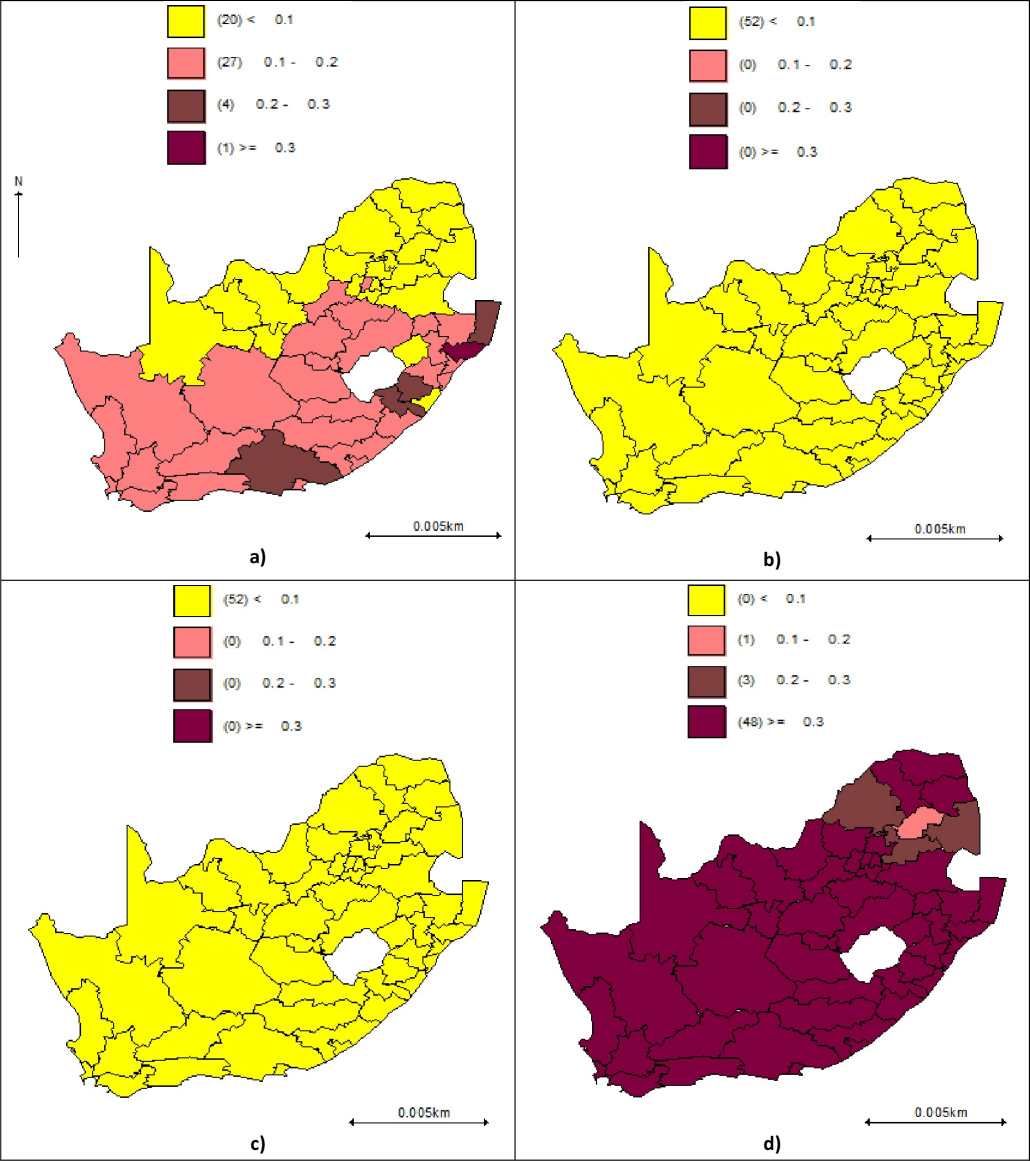
The spatial distribution of the individual, household and district SAMPI covariate-adjusted smoothed estimated spatial odds for each of the four disease-specific risks (HBP (a), IHD (b), stroke (c) and dyslipidaemia (d)) in the South African National Health and Nutrition Survey, 2012. Estimated from model B spatial odds are indicative of excessive risk of a CVD in a given district.

The distribution of disease-specific spatial odds after adjusting for individual, household and the district-level SAMPI are shown in Fig 4. As before, most of the variations in the four diseases appeared to be removed, except for HBP which still has pockets of districts with higher excess risk in south-eastern parts.

## Discussion and conclusion

This paper analysed the sub-national co-morbidity of two major cardiovascular diseases: ischemic health disease (IHD), stroke and two major CVD risk conditions; hypertension and dyslipidaemia and their association to purported risk factors including household and district poverty in South Africa. This was done within an application of joint Bayesian spatial modelling that provided a better understanding of the health conditions by pooling data within and across the four conditions in a unified way. The observed prevalence data of the studied CVDs varied across the districts, with higher rates of hypertension in the south-eastern parts of the country, while IHD and dyslipidaemia had higher rates in the mainly north-eastern parts. Stroke was evenly distributed across the country. These variations dissipated when most of the purported individual and household risk factors and area poverty were included within a multivariate spatial modelling.

There were differences between the prevalence of high blood pressure (HBP) in urban formal areas compared to that in rural informal areas, with higher rates in the urban areas. This finding confirms results in Kandala et al [10,11] where both observed and estimated prevalence of hypertension and IHD to be high in the relatively urban areas), and low in the north east (as we found mostly rural). Looking at district level, the pattern observed in this study shows a high prevalence of IHD and HBP in the metropolitan and neighbouring areas. A study by Ibrahim and Damasceno [37] on hypertension in developing countries found a strong spatial association between hypertension mortality and social deprivation. These findings highlight the need to be vigilant about cardiovascular risk both in areas of extreme social deprivation and in more affluent metropolitan areas.

After adjusting for age, sex, education, urban-dwelling, ethnicity, smoking, obesity and socioeconomic quintile, we still found that estimated risks of stroke, hypertension and IHD were elevated in sixteen districts mostly in KwaZulu Natal and Mpumalanga compared to all the other districts mostly in Western Cape. This is in contrary to findings by Kandala et. al [10,11] that risks of these CVDs were more in districts in the south western parts of the country. The risk of dyslipidaemia was highly prevalent in Central and top |North-Eastern corridors of the South Africa, similar to Kandala et al. [10,11] findings. In terms of spatial clustering related to hypertension and risk factors, Zhou et al., [12] using Kulldorff spatial scan statistic identified localized clustering of overweight in a rural KwaZulu Natal area. This was however nullified when purported individual risk factors were modelled. However, the overall smoothed spatial risk of CVD did not change significantly. This therefore implies that some of the well-known risk factors partially explain small-area association in the risk of CVDs.

The spatial variation of CVDs across space could be attributed to shared lifestyle related behaviours. Overall, prevalence of hypertension (HBP) increased significantly with increasing age (mainly over 45 years) and those with no schooling. This agrees with findings by Ibrahim and Damasceno [37] and Sarki et al [7] that increasing prevalence of hypertension in developing countries is often caused by ageing of the population, changes in dietary habits, social stress as a result of urbanized patterns. Sarki et al [7] concluded that on average, about 1 in 3 adults in the developing world is hypertensive. They also observed that prevalence estimates significantly varied across the geographical regions but did not find significant differences in relation to sex. Similar to this study, persons who have no formal education are obese, and live in urban areas were also more likely to be hypertensive, compared with those who were educated, of normal body mass index, and rural dwellers respectively.

Diabetes had a strong positive association with three of the four of the cardiovascular diseases namely HBP, IHD and dyslipidaemia. It significantly increased the risk of HBP by almost 3 times [OR 2.819 (CI 2.201–3.627)]. Its association with stroke was positive, though not significant. This is consistent with evidence that individuals with diabetes are at high risk of atherosclerotic cardiovascular diseases and dyslipidaemia [38]. As diabetes is treatable, timely treatment could reduce the risk of heart disease and stroke and development of cardiovascular disease as the risk doubles when patients have both hypertension and diabetes.

The prevalence of CVDs is high and varies geographically across the country. Moreover, of concern, the extraordinarily high levels of risks of CVDs in poor rural health districts with the exception of high risks of IHD and dyslipidaemia in the city of Cape Town and Nelson Mandela Bay. The risks of CVDs are also high among those with no schooling or with primary education and in the advanced age groups.

These results have pointed to possible co-occurrence of the four CVDs at the district levels in South Africa. The inference of this finding to public health policy implies compounded burden of disease and costs that the local governments must bear. This may adversely impact CVD-related deaths and repeated hospitalizations as suggested by Ford et al [39]. Though, the present study did not analyse the prevalence of having 2 or more of the CVDs and associated factors, a number of studies across different populations and settings have found the occurrence of multiple CVD risk factors more prevalent; for example, among people aged 50 years and over in China, Ghana, Mexico, India, Russia and South Africa [40]; among urban slums dweller in Kenya [41], and in semi-urban South Africa [12] and among middle-aged and older immigrants in Australia [42].

The presence of both CVD co-occurrence at the district level and multiple risk factors has negative implications for CVD outcomes. A focus on implementing and intensifying interventions aimed at controlling these risk factors could have a positive effect on reducing co-occurrence of CVDs, either at the individual or sub-national levels. A point can be made for both inclusive and differentiated approaches for effective NCD prevention and control in South Africa. Haregu et al [41] and Li and Wright [43] suggested an integrated but segregated intervention. On the basis that many risk factors are associated with many major chronic diseases and since these risk factors act synergistically rather than additively [43] an integrated approach would be more beneficial as it may positively impact on many chronic conditions. The South African government has taken legislative steps to reduce the occurrence of unhealthy diets by introducing appropriate food labelling and sodium content [44]. However, further research is needed to expound on the impact that these interventions have had on NCD outcomes and associated with unhealthy food consumption patterns and behaviours (i.e. consumption of foods with high fat, sugar and salt content).

### Study limitations

The findings in our study are subject to some limitations. Firstly, it is a cross-sectional study and some variables, like, smoking status, stroke and the IHD status, were self-reported and thus may have brought about misclassifications. The other study outcome measures like HBP and dyslipidaemia incorporated both self-reported history of chronic NCDs, and physical and clinical examination such as measures of blood pressure and cholesterol. The self-reported measures are subject to participant social desirability and recall biases. Misclassification biases may be especially marked with the variable self-reported history of NCDs. The self-reported nature of the CVD data could lead to inaccurate measurements of the true prevalence of the CVD conditions. Tenkorang [45] note that its is important to complement self-report data with biometric data, as the latter markedly improves the accuracy of parameters estimated from populations at the individual level. By obtaining participants’ biomarkers in LDL and HDL the SANHANES-I attempted to address some of these limitations. Unfortunately, more special investigations such as electrocardiograms (ECGs) or gastroscopies were not done to try and verify some of the self-reported measures. Mentz [46] work on sensitivity, specificity and accuracy between clinical and self-reported measures found that measures within NHANES (a survey following similar design to SANHANES) sample were ‘good to excellent’. Taylor [47] noted that while self-reported measures are consistent over time, the differences between the self-reported measures and the actual clinical measurements could be explained by the technical aspects associated with clinic measurements. We have found very low sensitivity and high specificity values for our data on self-reported high blood pressure and dyslipidaemia, which is consoling as by using the measured markers we have captured the cases that could have been missed based on self-reporting.

The analysis and the resulting models depend upon the extent and validity of biological, physical and clinical examination including blood sugar levels, blood pressure and cholesterol. A substantial number of participants had these data missing. This may have affected the results, particularly if those who declined these measurements had different lifestyle behaviours compared to those who participated. We did not impute for missing data, so we could not ascertain effect of missing data on the overall results and subsequent conclusions. We also analysed a single cross-sectional data set, thus the results give a picture of the CVD situation in 2012 in the country. Apart from missing data, the study could not account for some important other equally important predictors of CVDs. Lack of these data could have confounded our findings.

The design of the SANHANES was to generate a sample that was representative at national and regional levels, but not at a lower geographic level, say districts. As in many similar surveys, the data at lower administrative levels may not have been systemically covered sufficiently. Reliable estimates as such lower levels have been shown to be highly associated with number of observations falling in these lower levels [48]. As subnational estimates are more than ever needed for local level planning in South Africa, the best statistical approach would be to design the survey to be representative at the desired geographic level. This could lead to increasing the sampling size of the next SANHANES or a different survey, which could lead to a very costly survey. Alternatively, a systematic evaluation and validation of a number of spatial models for generating CVD estimates at higher resolutions (lower administrative levels) could be undertaken as in the Subnational Estimates Working Group of the HIV Modelling Consortium (2016) [49]. However, in using the spatial statistics methods that employ local and global smoothing to borrow strengths across neighbouring areas, our approach has minimally mitigated the adverse effect of the under-powered data at the district level.

## Conclusions

The study has confirmed the importance of established individual and ecological risks factors for two major cardiovascular diseases: ischaemic heart disease, stroke and two major CVD risk conditions: hypertension and dyslipidaemia in South Africa. Thus, improving on these risk factor profiles in the South African population using timely and targeted interventions could lower the prevalence of these conditions and decrease their co-morbidity. Evidence of sub-national variations in the four studied conditions provides insights into optimal interventions routes for the prevention and control of CVDs and the associated risk factors. Further research is needed to elucidate the additional correlates and possible impacts of co-occurrence of multiple NCD risk factors.

As a conclusion, the finding that diabetes is a major shared risk factor for HBP, IHD and dyslipidaemia and to some extent stroke. Coupled with the geographical variation of the outcomes, these findings have impact of health policy interventions. Studies have shown physical inactivity is modifiable major risk factor for insulin resistance and cardiovascular disease. Thus, policies and intervention prompting exercising and losing weight can prevent or delay the onset of type 2 diabetes. This could reduce the incidence of high blood pressure (hypertension) which is a risk for heart attack and stroke. These measures could be implemented in conjunction, with health promotion campaigns to reduce smoking. Smokers whether or not they have diabetes are at higher risk for heart disease, stroke, hypertension and high blood cholesterol. Thus interventions to reduce smoking could bear positive results in the fight against the emergence of CVDs. Local government also need to design and put in place policies aimed at increasing uptake of healthy diets, reducing harmful alcohol consumption and other harmful lifestyle behaviours.

## Supporting information

S1 Table Posterior median odds ratios (95% CI) of the four cardiovascular conditions (HBP, IHD, stroke and dyslipidaemia) across selected individual and household covariates: South Africa 2012. (DOCX)

S1 Fig The individual and household covariate-adjusted estimated spatial odds for the four cardiovascular diseases (HBP (a), IHD (b), stroke (c) and dyslipidaemia (d)) in the South African National Health and Nutrition Survey, 2012. Spatial odds are indicative of excessive risk of a CVD in a given district.
(TIF)
