# Supplementary material for: Small-area variation of cardiovascular diseases and select risk factors and their association to household and area poverty in South Africa: Capturing emerging trends in South Africa to better target local level interventions
Source: PLoS One. 2020 Apr 22;15(4):e0230564. doi: 10.1371/journal.pone.0230564 (PMC7176123; doi:10.1371/journal.pone.0230564)
Supplement: S1 Table — (DOCX) [file pone.0230564.s001.docx]

**S1 Table. Posterior median odds ratios (95% CI) of the four cardiovascular conditions (HBP, IHD, Stroke and Dyslipidaemia) across selected individual and household covariates: South Africa 2012.**

|  | **High Blood Pressure(HBP)** | **Ischaemic Heart Disease(IHD)** | **Stroke** | **Dyslipidaemia** |
| --- | --- | --- | --- | --- |
| **Population Group** | **Adjusted Odds ratios (95% CI)** | **Adjusted Odds ratios (95% CI)** | **Adjusted Odds ratios (95% CI)** | **Adjusted Odds ratios (95% CI)** |
| **Sex** |  |  |  |  |
| Female | 1.0 | 1.0 | 1.0 | 1.0 |
| Male | 0.8 (0.7-1.0) | 0.7 (0.5-0.) | 0.9 (0.6-1.3) | 0.8 (0.7-0.9) |
| Age(Years) |  |  |  |  |
| 15-24 | 1.0 | 1.0 | 1.0 | 1.0 |
| 25-34 | 1.7 (1.3-2.2) | 2.1 (1.3-3.5) | 2.2 (0.9-5.6) | 1.1 (0.9-1.4) |
| 35-44 | 3.8 (2.9-4.9) | 3.3 (2.1-5.3) | 4.6 (2.1-11.4) | 1.3 (1.1-1.6) |
| 45-54 | 9.1 (7.0-11.7) | 3.5 (2.2-5.6) | 4.7 (2.1-11.5) | 1.6 (1.3-2.0) |
| 55-64 | 16.0 (12.1-21.2) | 4.3 (2.7-7.0) | 8.0 (3.6-19.6) | 1.5 (1.2-1.8) |
| 65+ | 25.6 (18.6-35.2) | 5.0 (3.1-8.3) | 11.6 (5.2-28.9) | 1.3 (1-1.6) |
| **Education** |  |  |  |  |
| No schooling | 1.0 | 1.00 | 1.00 | 1.00 |
| Primary school | 1.1 (0.8-1.) | 0.9 (0.6-1.4) | 1.2 (0.7-2.1) | 1.169 (1.0-1.5) |
| Secondary school | 1.1 (0.8-1.5) | 0.8 (0.5-1.2) | 1.2 (0.7-2.2) | 1.173 (1.0-1.5) |
| Matric | 0.0 (0.7-1.4) | 0.5 (0.3-0.9) | 1.0 (0.4-2.1) | 1.1 (0.8-1.4) |
| Higher education | 1.203 (0.8-1.8) | 0.7 (0.3-1.3) | 1.3 (0.6-3.2) | 0.9 (0.6-1.2) |
| **Race** |  |  |  |  |
| African | 1.00 | 1.00 | 1.00 | 1.00 |
| White | 0.9 (0.6-1.6) | 1.6 (0.8-3.1) | 1.0 (0.2-2.6) | 3.0 (1.9-4.7) |
| Coloured | 1.3 (1.0-1.7) | 0.7 (0.5-1.1) | 1.1 (0.6-1.9) | 1.0 (0.8-1.2) |
| Indian/Asian | 0.7 (0.4-1.0) | 0.7 (0.4-1.3) | 0.4 (0.1-1.1) | 1.4 (1.0-2.0) |
| **Locality** |  |  |  |  |
| Urban formal | 1.00 | 1.00 | 1.00 | 1.00 |
| Urban informal | 0.8 (0.6-1.1) | 0.5 (0.3-0.8) | 0.7 (0.3-1.5) | 0.8 (0.6-1.0) |
| Rural informal(Tribal) | 0.7 (0.5-0.9) | 0.9 (0.6-1.4) | 1.2 (0.7-2.0) | 0.9 (0.7-1.1) |
| Rural formal(Farms) | 0.9 (0.7-1.19) | 0.6 (0.4-1.0) | 1.0 (0.5-1.7) | 1.0 (0.8-1.3) |
| **BMI*** |  |  |  |  |
| Normal | 1.00 | 1.00 | 1.00 | 1.00 |
| Underweight | 0.7 (0.5-0.9) | 1.3 (0.8-2.1) | 1.1 (0.5-2.3) | 0.6 (0.5-0.8) |
| Overweight | 1.7 (1.4-2.0) | 1.1 (0.8-1.5) | 1.1 (0.8-1.8) | 1.9 (1.7-2.2) |
| **Diabetes** |  |  |  |  |
| **No** | 1.00 | 1.00 | 1.00 | 1.00 |
| **Yes** | 2.8 (2.2-3.6) | 1.8 (1.3-2.4) | 1.4 (0.9-2.1) | 1.7 (1.3-2.1) |
| **Smoking** |  |  |  |  |
| Never | 1.00 | 1.00 | 1.00 | 1.00 |
| Smoker | 1.1 (0.9-1.3) | 1.0 (0.7-1.4) | 1.0 (0.6-1.6) | 1.0 (0.9-1.2) |
| **Alcohol use** |  |  |  |  |
| Never | 1.00 | 1.00 | 1.00 | 1.00 |
| Drinker | 1.1 (0.9-1.3) | 0.9 (0.7-1.3) | 1.3 (0.8-2.0) | 0.8 (0.6-0.9) |
| **Wealth quintiles (households survey)** |  |  |  |  |
| QI (Lowest) | 1.00 | 1.00 | 1.00 | 1.00 |
| QII | 1.0 (0.8-1.2) | 0.8 (0.6-1.2) | 1.161 (0.6-2.2) | 0.8 (0.7-1.0) |
| QIII | 0.9 (0.7-1.2) | 1.7 (1.-2.5) | 2.26 (1.3-4.1) | 0.7 (0.6-0.9) |
| QIV | 0.9 (0.7-1.2) | 1.1 (0.7-1.7) | 2.072 (1.1-4.0) | 0.9 (0.7-1.1) |
| QV(Highest ) | 1.2 (0.9-1.7) | 0.8 (0.5-1.4) | 0.9572 (0.4-2.2) | 1.4 (1.1-1.8) |
| **normal weight (BMI 18.5 > 25.0) vs. underweight (BMI < 18.5), overweight/obese(BMI ≥ 25)* | | | | |
