# Supplementary figures and images for: Small-area variation of cardiovascular diseases and select risk factors and their association to household and area poverty in South Africa: Capturing emerging trends in South Africa to better target local level interventions
Source: PLoS One. 2020 Apr 22;15(4):e0230564. doi: 10.1371/journal.pone.0230564 (PMC7176123; doi:10.1371/journal.pone.0230564)

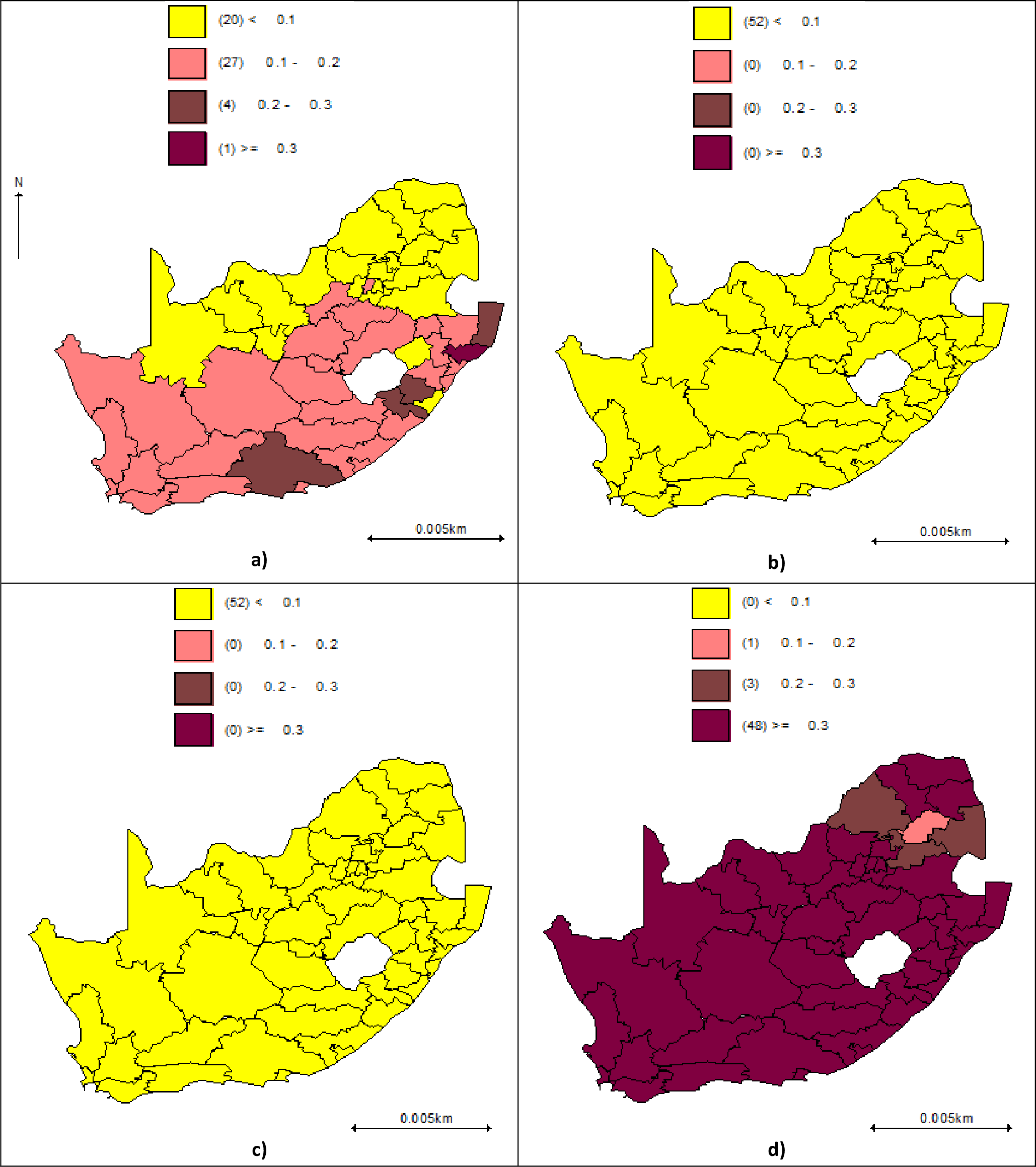

Supplement: S1 Fig — The individual and household covariate-adjusted estimated spatial odds for the four cardiovascular diseases (HBP (a), IHD (b), stroke (c) and dyslipidaemia (d)) in the South African National Health and Nutrition Survey, 2012. Spatial odds are indicative of excessive risk of a CVD in a given district. (TIF) [file pone.0230564.s002.tif]
